# Relatively Low Prevalence of Peripheral and Placental *Plasmodium* Infection at Delivery in Bangui, Central African Republic

**DOI:** 10.1155/2011/434816

**Published:** 2011-12-22

**Authors:** Alexandre Manirakiza, Eugène Serdouma, Djibrine Djalle, Georges Soula, Remi Laganier, Nestor Madji, Methode Moyen, Alain Le Faou, Jean Delmont

**Affiliations:** ^1^Institut Pasteur de Bangui, Avenue Pasteur, P.O. Box 923, Bangui, Central African Republic; ^2^Centre de Formation et de Recherche en Médecine et Santé Tropicales, Faculté de Médecine Nord, Boulevard Dramard, 13015 Marseille, France; ^3^Reproductive Health and Malaria Program Division, Ministry of Public Health, Population and AIDS Control, Bangui, P.O. Box 883, Bangui, Central African Republic; ^4^Faculty of Health Sciences, University of Bangui, P.O. Box 1383, Central African Republic; ^5^Hôpital de Brabois Adultes, CHU de Nancy, 54511 Vandœuvre-lès-Nancy Cedex, France

## Abstract

*Introduction*. The aim of this study was to estimate the prevalence of malaria among women giving birth in Bangui. Association between sociodemographic characteristics of those women and malaria, as well as prevention compliance (use of intermittent preventive treatment with sulfadoxine-pyrimethamine (IPTsp) and insecticide-treated bed nets (ITNs)), was analyzed. *Methods*. During September 2009, a survey was conducted on 328 women who gave birth at two main maternities of Bangui. Information was obtained by standardized questionnaire about sociodemographic criteria, IPTsp, other antimalarial treatment, and use of bet nets. Smears prepared from peripheral and placental blood were analysed for malaria parasites. *Findings and Discussion*. Positive results were found in 2.8% of thick peripheral blood smears and in 4.0% of placental slides. A proportion of 30.5% of the women had received at least two doses of IPTsp during the current pregnancy. Only a proportion of 42.4% of this study population had ITNs. Multigravid women were less likely to use IPTsp and ITNs. However, use of IPTsp was associated with personal income and secondary or university educational status. Hence, although this relatively prevalence was observed, more efforts are needed to implement IPTsp and ITNs, taking into account sociodemographic criteria.

## 1. Introduction

Every year, it is estimated that tens of thousands of pregnant women in malaria-endemic areas are infected with *Plasmodium falciparum *[1]. Frequently, placental infection occurs, owing to the accumulation of *P. falciparum-*infected erythrocytes in the intervillous space, despite the absence of parasites in peripheral blood [2]. The complications of malaria during pregnancy are maternal anaemia, preterm delivery, and low birth weight of newborns, which increase perinatal morbidity [1, 3, 4]. The World Health Organization (WHO) recommends intermittent preventive treatment with sulfadoxine-pyrimethamine (IPTsp) during pregnancy, with at least two doses after quickening (18–20 weeks) not more frequently than monthly, use of insecticide-treated bed nets (ITNs) and prompt treatment of clinical malaria [5]. Intermittent preventive treatment consists of delivering a curative treatment dose of an antimalarial at predefined intervals, regardless of the parasitological status of the woman, and the efficacy of this protocol has been demonstrated in a number of malaria-endemic countries [5–8].

Placental *Plasmodium* screening in the Central African Republic (CAR) in 1990 showed a rate of 37.1% in women who had been given chemoprophylaxis with chloroquine [9]. In 2006, the Ministry of Health of the CAR adopted and implemented the new WHO recommendations for malaria prevention during pregnancy. The aim of the study reported here was to estimate the prevalence of malaria in thick peripheral blood smears and placental blood from a sample of women who gave birth during September 2009 at two main maternities of Bangui, the capital of CAR. Secondly, we assessed the women's coverage rates with the three components of the WHO package [10] for malaria management during the current pregnancy and identify pregnant women characteristics associated with IPTsp and ITNs.

## 2. Methods

### 2.1. Study Setting

We conducted a cross-sectional study in the two main maternity clinics of Bangui, the Castors health Centre and the “Communautaire” Hospital, in September 2009. The geographic coordinates of Bangui is 7°.00′ north and 21°.00′ east. The climate is tropical, and rainfall peaks are observed from April to November, and temperature ranges from 19°C to 32°C. The main parasite is *Plasmodium falciparum,* and malaria transmission is perennial with peaks during the rainy season, but no data on the intensity of malaria transmission (entomological inoculation rates) is available in CAR. Malaria represents more than 40% of morbidity in Bangui, as well as in other CAR areas [11].

The Castors Health Centre and the “Communautaire” Hospital provide antenatal and delivery services and have established programmes for the prevention of malaria and other infectious diseases, such as mother-to-child transmission of HIV infection. Each year, it is estimated that 12,000 women deliver at those centres, representing 70% of all women who deliver in Bangui. All women are screened for malaria by microscopic analysis of 4% Giemsa-stained thick blood smears during visits to the antenatal clinics, performed either at the same health centre or at one of the national reference biomedical laboratories (the National Laboratory for Clinical Biology and Public Health and the Institut Pasteur de Bangui), depending on each woman's choice. Antimalarial treatment is prescribed on the basis of clinical symptoms if the woman cannot afford the laboratory fees immediately.

For asymptomatic women, IPTsp is given free of charge as directly observed therapy during the antenatal visit. Administration of two doses is recommended for HIV-negative women and three doses for HIV-positive women.

### 2.2. Study Population, Enrolment, and Data Collection

All women from whom we obtained written informed consent were eligible for the study immediately after delivery. A study midwife administered a standardized questionnaire to record sociodemographic data (age, residence area, literacy, number of gravidities, and monthly income), HIV serological status, intake of antimalarial medications (IPTsp and other antimalarial prescriptions), and bed net use.

Malaria prevalence of 30% at the time of childbirth was used as a proxy to calculate the sample size in this study. Thus, a number of 323 women was necessary assuming 90% power at 5% significance level.

Because of lack of an ethical committee in the CAR, this project was reviewed and approved by an *ad hoc* scientific committee of the University of Bangui in charge of validating scientific study protocols in the CAR. The study was conducted under a collaborative agreement with the University of Marseille, France.

### 2.3. Laboratory Analyses

Peripheral venous and placental blood from each woman was used to prepare thick blood films. Placental blood was obtained as follows: immediately after delivery, the paracentric side of the maternal placenta was cleaned with sterile water and incised, and thick blood films were prepared from a droplet collected by aspiration through a 21-gauge needle attached to a 5-mL syringe, as described previously [12, 13].

The thick smears were air-dried and stained with 4% Giemsa. At each of the two study sites, an experienced microscopist immediately examined the stained smears by light microscopy (×100 oil immersion) to detect asexual forms of *P. falciparum* malaria parasites. On peripheral blood slides, malaria parasites were counted in 200 leukocytes, and the parasite density per microlitre of blood was estimated as the number of parasites counted multiplied by 40 under the assumption of a leukocyte count of 8000/*μ*L of blood. For both types of blood film, a result was considered to be negative if no parasites were detected per 200 leukocytes. Women with a positive peripheral blood result were given antimalarial treatment (either artemether-lumefantrine or quinine, depending on a clinical evaluation and individual tolerance).

All the slides were analysed twice at the Biomedical Laboratory of the Institut Pasteur de Bangui.

### 2.4. Data Analysis

Data were double-entered into EpiInfo software version 3.5.1, and the database was checked and data entry errors corrected with the EpiInfo software “data compare” utility for finding differences between two tables. Statistical analysis was conducted with Stata 8.0 and MedCalc v11.6.1. The association between sociodemographic criteria and malaria, use of IPTsp and ITNs was examined using the chi-squared test, and association between those variables was tested by calculating the odds ratios (ORs). The estimates were achieved at 95% interval confidence. Differences between proportions ≤5% were considered as significant.

## 3. Results

### 3.1. Study Population Characteristics

Overall, 328 pregnant women delivering at the two health centres were included in the study: 168 (51.2%) at the Castors and 160 (48.8%) at the “Communautaire” Hospital. A large proportion of them reside in Bangui city (96.0% or 314/328). The mean age was 23 years (range, 14–39 years), and 33.8% were aged less than twenty years. A proportion of 33.0% (108/328) were primigravid. Most of those women do not have any personal monthly income (57.9%), but the majority of them have at least secondary educational status. 

HIV infection had been screened for 58.2% (191/328) of the population, resulting an infection prevalence of 9.1% (15/164; 27 HIV results could not be cross-checked on the antenatal clinic cards).

Sleeping daily under bed net was reported by 81.5% (95% CI, [77.3–85.7]) of the women, and 42.4% (95% CI, [36.8–48.0]) had ITNs.

### 3.2. Attendance at Antenatal Clinics

**Figure 1:**
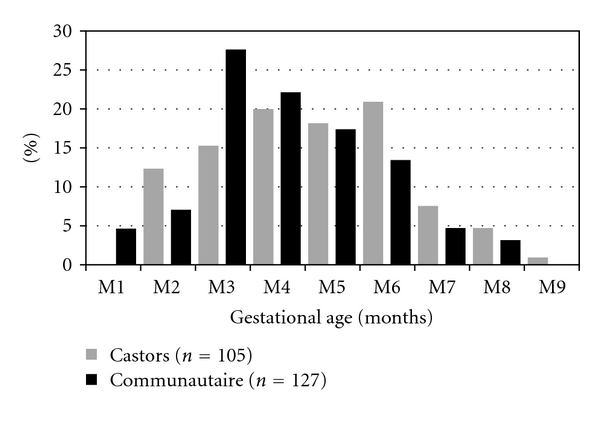
First antenatal clinic attendance according to gestational age, September 2009, Central African Republic.

At the two study sites, checking of antenatal clinic cards showed that 93.3% (95% CI, [90.8–95.8]) of the women had presented at least one antenatal visit. Less than one fourth of the women (24.1%; 95% CI, [19.3–28.9]) had attended an antenatal clinic during the first trimester of pregnancy, while the majority (55.6%; 95% CI, [50.0–61.2]) had attended a clinic during the second trimester. Overall, 35.6% (95% CI, [31.1–40.1]) completed four antenatal visits. The distribution of first antenatal attendance according to gestational age at each study site is shown in Figure 1.

### 3.3. Any Antimalarial Treatment during the Pregnancy

**Figure 2:**
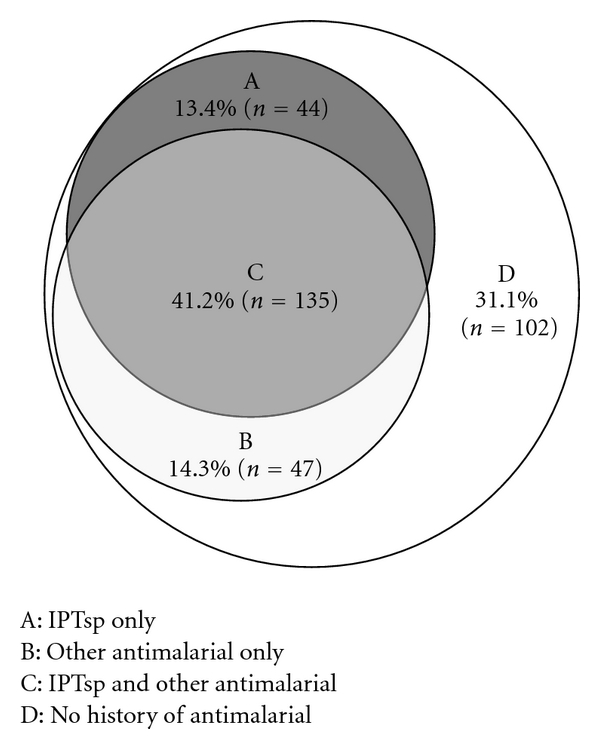
Distribution of antimalarial (IPTsp *or any other antimalarial*) use during pregnancy, September 2009, Central African Republic. IPTsp: intermittent preventive treatment with sulfadoxine-pyrimethamine.

Of the women who received IPTsp, 75.4% (95% CI, [69.1–81.8]) were given curative prescriptions of other antimalarial drugs, independently of the timing of IPTsp doses (Figure 2). The antenatal clinic cards of 182 women (55.5%; 95% CI, [50.1–60.1]) showed a history of at least one curative treatment for malaria. Of these women, 27.0% had been prescribed an antimalarial drug two or three times. Although 228 antimalarial prescriptions were recorded on antenatal clinic cards during the current pregnancy, only 56 laboratory results were positive out of the total 73 blood smears analysed. The antimalarial drugs prescribed were quinine (66.7% or 152/228; 95% CI, [60.6–72.8]), artemisinin-based combinations (15.5%; 95% CI, [10.7–20.1]) and artemisinin monotherapy (18.0%; 95% CI, [13.0–23.0]).

**Figure 3:**
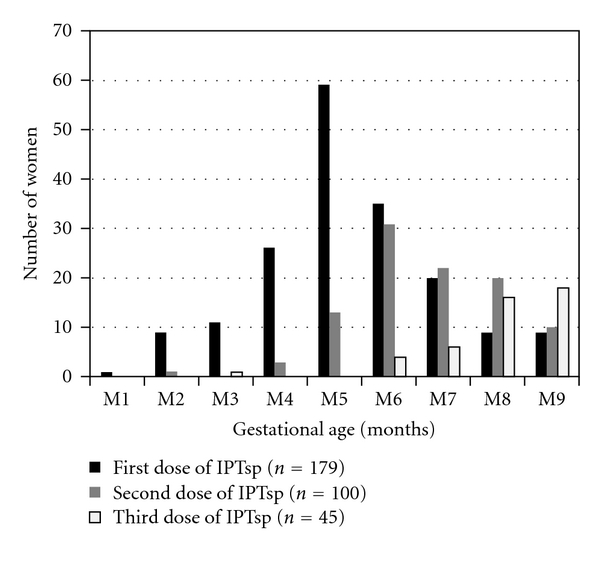
Distribution of intermittent preventive treatment with sulfadoxine-pyrimethamine (IPTsp) doses by gestational age, September 2009, Central African Republic.

At least one dose of IPTsp was recorded for 54.6% (95% CI, [49.2–60.0]) of our study population; only 30.5% (95% CI, [23.8–37.2]) had received at least two doses. Most of these women (78.2%; 95% CI, [72.2–84.3]) had received the first IPTsp dose between the fourth and seventh months of pregnancy; however, 11.7% (95% CI, [7.0–16.4]) had received the first dose during the first trimester. Details of IPTsp administration are shown in Figure 3. 

### 3.4. Relation between Sociodemographic Characteristics and the Compliance to Malaria Prevention

Multigravid women, were less likely to use two doses of IPTsp (OR = 0.14; 95% CI, [0.08–0.24], *P* < 0.0001) and ITNs (OR = 0.16; 95% CI, [0.10–0.28], *P* < 0.001) compared to primigravid women. Use of IPTsp (two doses) was associated with lucrative activities (OR = 4.20; [2.55–6.92], *P* < 0.0001) and secondary or university educational status (OR = 2.22; 95% CI, [1.33–3.72], *P* = 0.002). Women with secondary or university educational status were also likely to use INTs (OR = 1.90; 95% CI, [1.20–3.01], *P* = 0.01). Details on association analysis between sociodemographic characteristics and those preventive tools use are shown in Table 1.

### 3.5. Results of Blood Smear Examinations

Overall, peripheral blood *P. falciparum* infection at delivery was found in 2.8% (95% CI, [1.0–4.6]) of peripheral blood and in 4.0% (95% CI, [2.0–6.0]) of placental blood. Of the women with placental malaria, 77.0% (10/13) declared not using any bed net and 53.8% (7/13) had not taken any antimalarial drug during pregnancy.

HIV serological status had no impact on these findings. IPTsp and ITNs use was found not to be associated with the women sociodemographical characteristics. Moreover, there is no statistically significant association between these laboratory findings and those characteristics.

## 4. Discussion

The prevalence of malaria among pregnant women in the CAR (2.8% of thick peripheral blood smears and 4.0% of placental slides) was lower than in other areas of intense malaria transmission, such as Gabon, where the rates were 34.4% in maternal blood and 53.6% in placental blood films [6]. A recent review [14] of randomized clinical trials and surveys on the efficacy of IPT showed overall placenta-positive rates ≥10%. Falade and coauthors [7] in Nigeria reported that the prevalence of placental parasitaemia was 10.5% in women given IPTsp and 17% in those with no chemoprophylaxis.

Three years after initiation of IPTsp in CAR, coverage with at least one dose of IPTsp was slightly more than 50%. Although WHO recommends two doses IPTsp for ≥80% of pregnant women [15], our estimate in this study was 30.5%. The low prevalence of placental malaria in our study is therefore probably due to the combination of IPTsp, other antimalarial drug, and use of ITNs. Hence, the relatively low prevalence of malaria at delivery is not surprising in Bangui. Indeed, a similar finding of malaria prevalence at the time of delivery is observed in Côte d'Ivoire, where Vanga-Bosson and coauthors report a prevalence of 4.8% of placental malaria, in an area where IPTsp coverage rate (≥2 doses) does not exceed 50% [16] and, in Thailand, where proportion of positive results for *P. falciparum* in maternal blood and in placental blood were estimated at 3.0% and 3.8%, respectively [17]. In Thailand, the authors report that this relative lower prevalence was due to antimalarial treatment with artemisinin derivatives. In our study, most of women were prescribed quinine or artemisinin-based combinations during the pregnancy. Indeed, those antimalarial drugs are actually the most efficacious on malaria [18].

Otherwise, our findings show that more than 80% of the women slept under a bed net. The efficacy of bed nets for preventing malaria is indisputable. Indeed, use of ITNs was found to reduce the incidence of uncomplicated malarial episodes in areas of stable malaria by 50% in comparison with no nets use and by 39% in comparison with untreated nets use [15].

In our study, the lower proportion of use of IPTsp and ITNs in multigravid women could be due to less health conscious of those women of their pregnancy. Inversely, the relative high proportion of use of IPTsp and ITNs in women with high level of education is due to the fact that higher educational status implies more health consciousness and is a factor influencing assimilation of health education programmes. Similar findings were reported in Cameroon and in Malawi [19, 20]. Women with salary or other personal income are also likely to be compliant with IPTsp and ITNs, possibly because they are able to afford health care fees.

The main limitation of our study is the only use of microscopic examination of blood smears. Even if, the microscopic analysis remains the standard detection of *Plasmodium *[21], submicroscopic infections are common during pregnancy [21, 22]. Hence, molecular methods (polymerase chain reaction or PCR) and rapid diagnostic tests (RDTs) provide finding approximately twice as many infections as microscopy [23–25]. However, determination of the possible impact of these submicroscopic infections to poor birth outcomes and maternal health is critical [26], and PCR is not feasible routinely [27]. To this end, microscopic examination to detect these infections is still essential, and implementation of RDTs use is challenging [21, 28].

## 5. Conclusion

Our results indicate that, although the recommended coverage rates of pregnant women with IPTsp and ITNs are not reached in Bangui, the prevalence of the main indicator of infection, placental malaria, is relatively low. The widespread presumptive prescription and consumption of antimalarial agents could indisputably be the cause of clearance of the existing peripheral and placental *Plasmodium* infection and decreased the risk of new infections over the pregnancy period. Indeed, symptoms suggestive of malaria are very frequent among pregnant women attending antenatal clinics, thus implying frequently unnecessary large use of antimalarial drugs [29]. For this purpose, strengthening national malaria control activities, taking into account prompt laboratory diagnosis and sociodemographic particularities, should contribute to the achievement of high coverage rate with the WHO preventive package components. Otherwise, cohort studies are needed to assess the real efficacy of IPTsp in preventing malaria during pregnancy.

##  Authors' Contributions

A. Manirakiza and J. Delmont conceived the study. Field data and blood samples analysis were achieved by A. Manirakiza, M. Moyen, D. Djalle, N. Madji and R. Laganier. Data analysis and interpretation were achieved by A. Manirakiza, J.Delmont, E. Serdouma, G. Soula, and Alain Le Faou. This draft was written by A. Manirakiza and J. Delmont. All authors read and approved this paper.

## Figures and Tables

**Table 1:** Association between some sociodemographic characteristics and the compliance to intermittent preventive treatment with sulfadoxine-pyrimethamine (IPTsp) and insecticide-treated nets (INTs) in Bangui, September 2009, Central African Republic.

		≥2 doses of IPTsp			Use of ITNs		
Criteria	*n*	%	OR [95% CI]	*P-*value	%	OR [95% CI]	*P-*value
Age (years)							
<20	111	36.9	1	NA	48.6	1	NA
20–24	105	27.6	0.65 [0.36–1.15]	0.18	40.0	0.70 [0.41–1.20]	0.25
25–29	74	27.0	0.63 [0.33–1.20]	0.21	29.7	0.44 [0.23– 0.83]	0.01
≥30	38	26.3	0.60 [0.26–1.38]	0.32	55.3	1.30 [0.62–2.73]	0.6
Gravidity							
1	108	58.3	1	NA	70.4	1	NA
≥2	220	16.8	0.14 [0.08–0.24]	<0.0001	28.6	0.16 [0.10–0.28]	<0.001
Monthly income							
No income	190	17.9	1	NA	46.3	1	NA
Salary or other personal income	138	47.8	4.20 [2.55– 6.92]	<0.0001	37.0	0.67 [0.43–1.06]	0.11
Educational status							
None or primary	130	20.8	1	NA	33.1	1	NA
Secondary or university	198	36.9	2.22 [1.33–3.72]	0.002	49.5	1.90 [1.20–3.01]	0.01
